# Three cases of non-infectious necrotizing stromal keratitis after corneal refractive surgery

**DOI:** 10.1093/jscr/rjad653

**Published:** 2024-03-15

**Authors:** Hu Chen, Ting Shen, Ling Tong Tan

**Affiliations:** Zhejiang Provincial People's Hospital, Ophthalmology, Gongshu District, Hangzhou City, Shangtang road No. 158, Hangzhou City, 310000, China; Department of Ophthalmologic Center, the Eye Hospital of the Second Affiliated Hospital of Zhejiang University, Hangzhou City, CN15268105588, China; Zhejiang Provincial People's Hospital, Ophthalmology, Gongshu District, Hangzhou City, Shangtang road No. 158, Hangzhou City, 310000, China

**Keywords:** FS-LASIK, SMILE, aseptic necrotizing stromal keratinitis, corneal refractive surgery

## Abstract

We reported three cases of aseptic necrotizing stromal keratinitis after corneal refractive surgery (two with small incision lenticule extraction and one with femtosecond laser-laser-assisted insitu keratomileusis). There were three young women who had undergone corneal refractive surgery had white aseptic infiltrating foci along or away from the stroma in both eyes or one eye on regular review, all of whom denied systemic disease or chronic ocular disease. Two patients were diagnosed with aseptic necrotizing corneal stromal inflammation, and one patient was diagnosed with delayed necrotizing corneal stromal inflammation. In our opinion, before corneal refractive surgery, medical history inquiry is very important. More attention should be paid to patients with vaccination history and foreign travel history. In addition, the possibility of delayed corneal stromal inflammation should be considered for patients with poor binocular corrected vision.

## Instruction

Coreal refractive surgery is the most commonly used myopia surgery. Small incision lenticule extraction (SMILE) and Femtosecond-Laser-In-Situ Keratomileusis (FS-LASIK) surgery are the most commonly performed corneal refractive surgeries. Corneal infiltration is an uncommon complication after corneal refractive surgery, but once it occurs, if not properly treated, it may lead to serious consequences. Although SMILE surgery has a smaller incision than FS-LASIK surgery, there is a possibility of corneal infiltration after either SMILE or FS-LASIK surgery.

## Case 1

**Figure 1:**
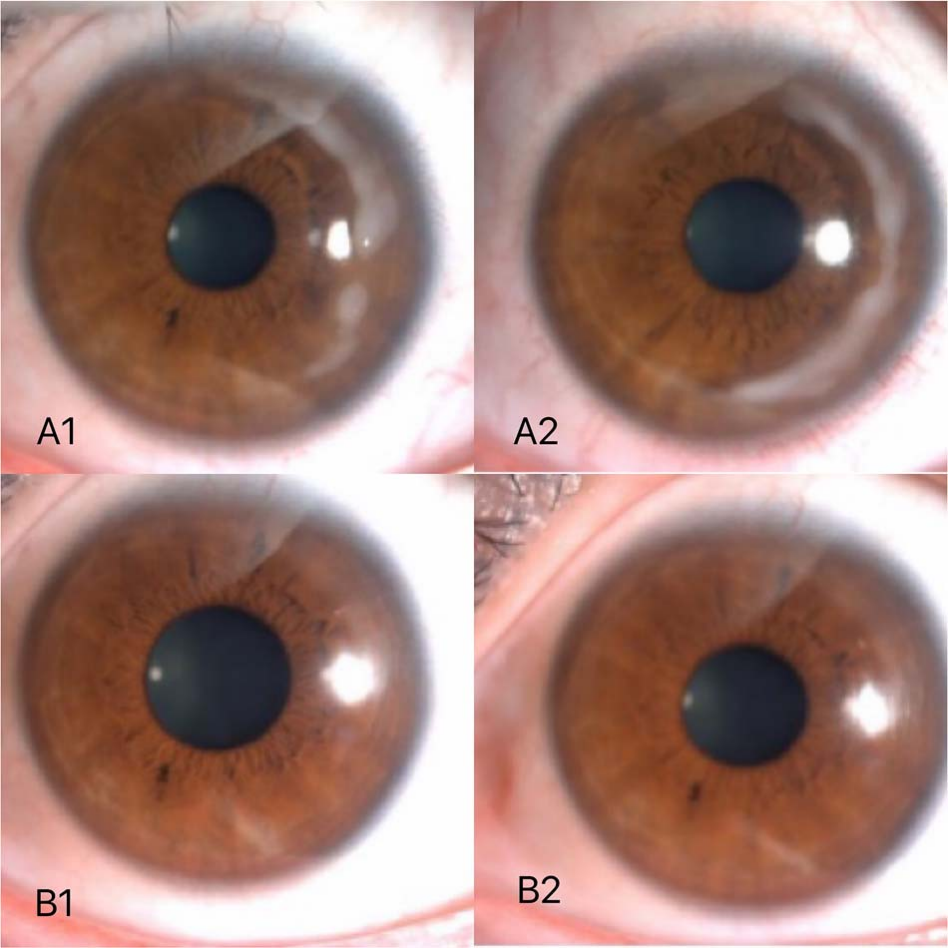
Three days after surgery, white stromal infiltration around the flap margin from 12 o’clock to 6 o’clock (A2) with intact but less regular epithelium. Examination of the left eye revealed the same lesion from 12 o’clock to 3 o’clock (A1). About 3 months after surgery,only a small amount of cloudy corneal opacity remained in both eyes (B1, B2).

**Figure 2:**
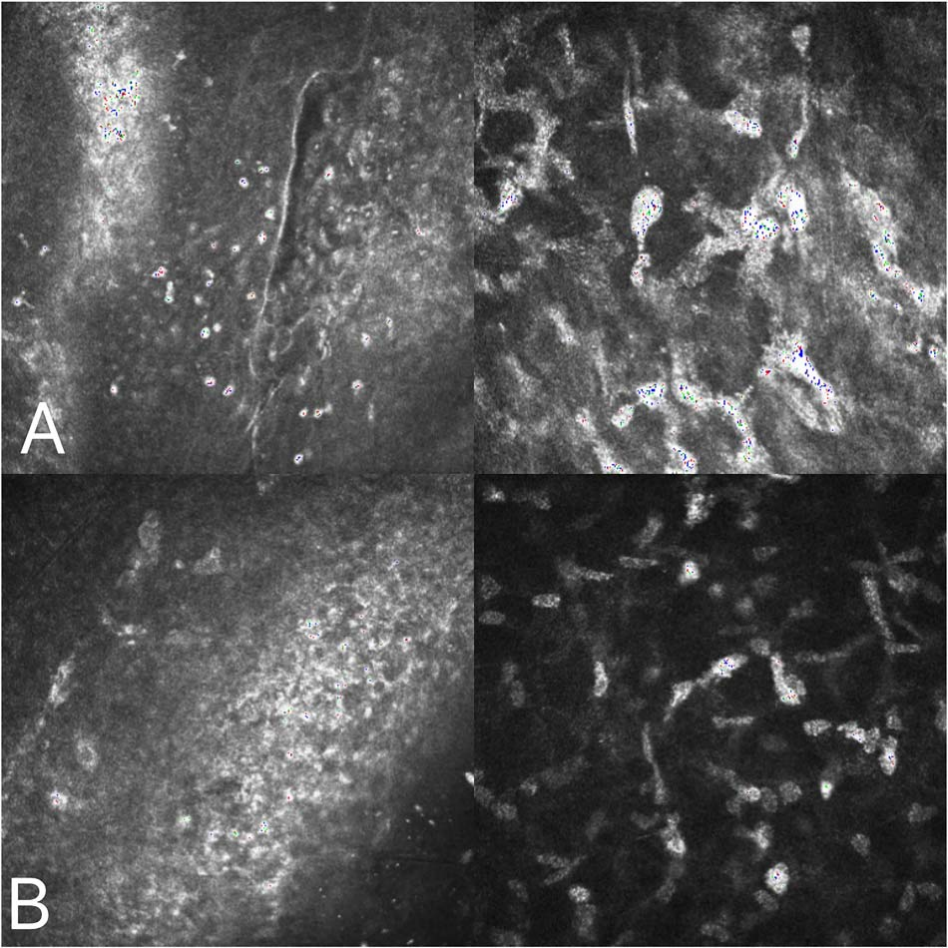
Corneal confocal microscopy on the third postoperative day: inflammatory cell infiltration was observed in the corneal epithelial layer (A), and a pine needle-like hyperreflective was observed in the superficial corneal stromal layer (B).

A 36-year-old female underwent preoperative corneal refractive evaluation at the Eye Hospital of the Second Affiliated Hospital of Zhejiang University in March 2022. Preoperative examination showed no significant abnormalities. The bilateral SMILE surgery went smoothly. Three days after surgery, the patient returned to the hospital for review. Slit-lamp examination of the right eye showed white stromal infiltration around the flap margin from 12 o’clock to 6 o’clock, with intact but less regular epithelium. Examination of the left eye revealed the same lesion from 12 o’clock to 3 o’clock (Fig. 1) and the patient complained of no apparent discomfort. The corrected visual acuity of both eyes was 20/20. Confocal microscopy of both eyes showed inflammatory cell infiltration was observed in the corneal epithelial layer, and a pine needle-like hyper-reflective was observed in the superficial corneal stromal layer. No hyphae were detected (Fig. 2). Considering the diagnosis of non-infective necrotizing interstitial keratitis. With oral and topical steroid treatment, the lesions gradually became limited in both eyes. At the latest follow-up, the patient had a good prognosis, with corrected visual acuity of 20/20 in both eyes, and only a small amount of cloudy corneal opacity remained in both eyes (Fig. 1).

## Case 2

**Figure 3:**
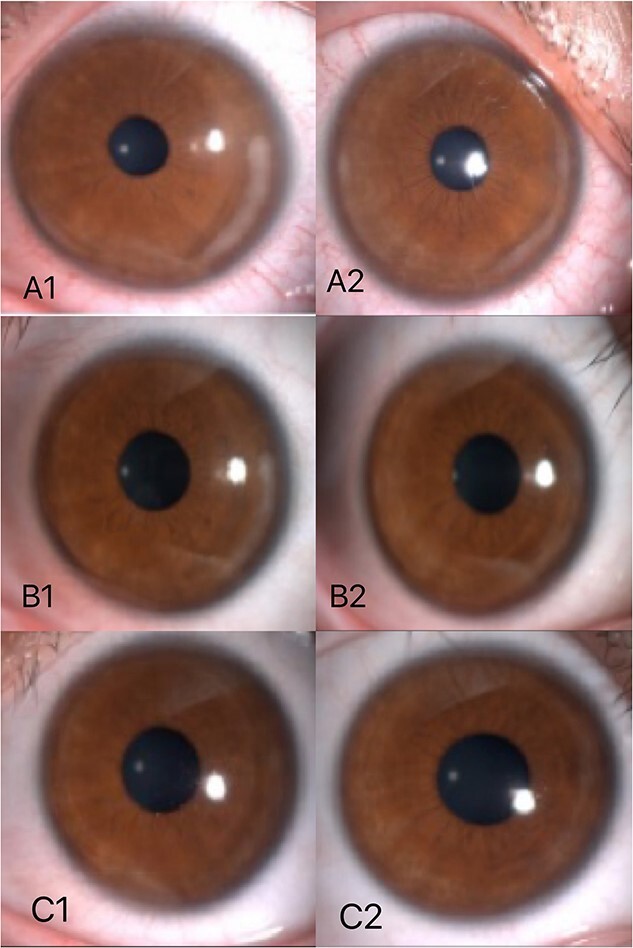
The third day after surgery, in the left eye, white infiltration was seen at the edge of the flap and the surrounding stromal within a range of 1 mm from 3 o ‘clock to 5 o ‘clock (A1). In the right eye, white infiltration was seen at the edge of the flap and the surrounding stromal from 1 o’clock to 5 o’clock and from 7 o’clock to 9 o’clock (A2). On the sixth day after surgery, lesions still existed in both eyes without further development and expansion (B1, B2). According to the last review,the infiltrations in both eyes almost disappeared (C1,C2).

**Figure 4:**
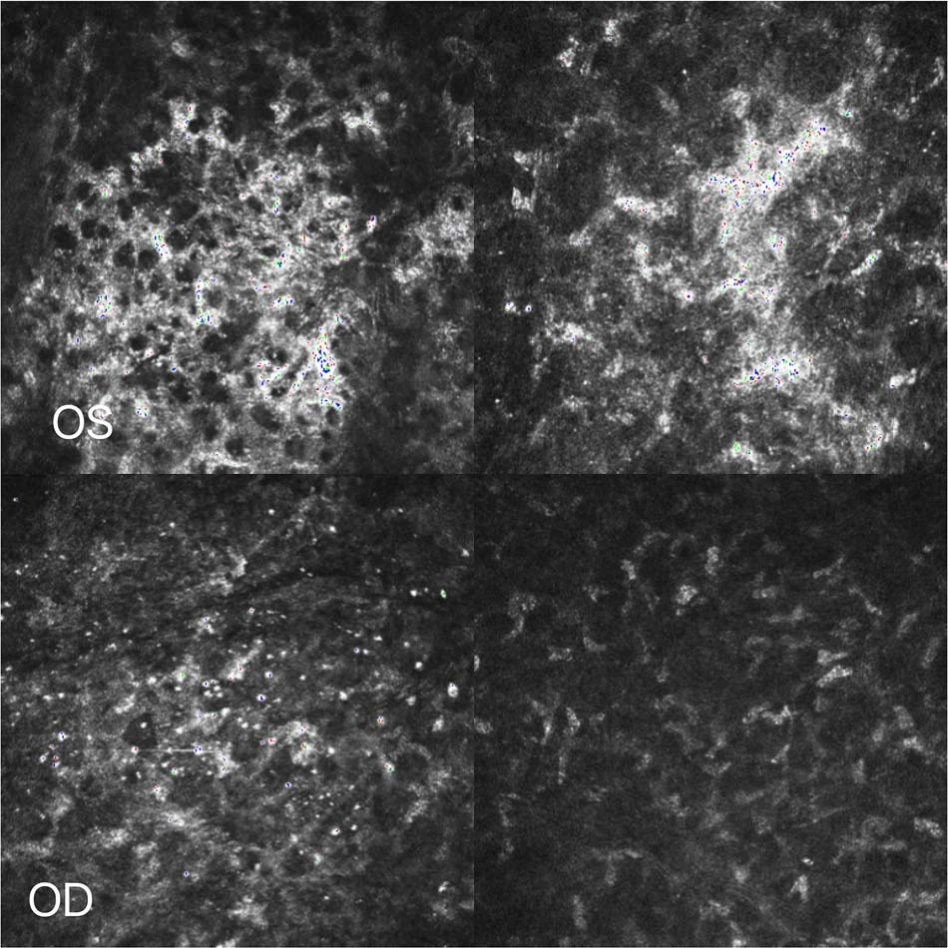
Corneal confocal microscopy: Inflammatory material deposition in the stromal layer was significantly reduced.

**Figure 5:**
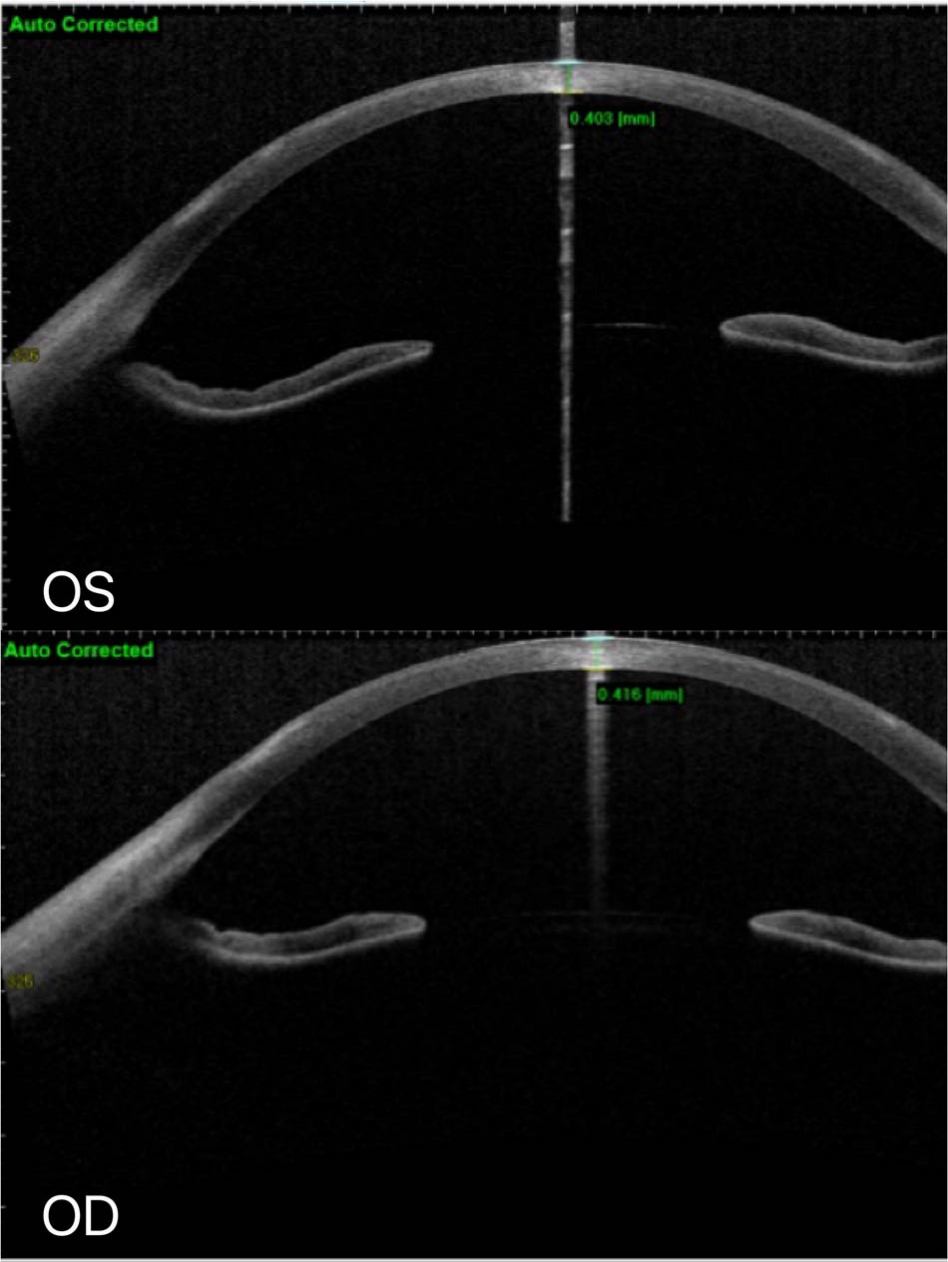
At the latest review, AS-OCT: No defect of corneal epithelium in both eyes, and white infiltration of superficial corneal stroma remained.

A 19-year-old young woman was admitted to the Second Affiliated Eye Hospital of Zhejiang University for corneal myopic refractive surgery in both eyes in July 2022. The patient denied systemic disease, and the relevant examination showed no obvious abnormalities. FS-LASIK surgery was successfully performed in both eyes. On the third day after surgery, under the slit lamp, in the left eye, white infiltration was seen at the edge of the flap and the surrounding stromal within a range of 1 mm from 3 o ‘clock to 5 o ‘clock. In the right eye, white infiltration was seen at the edge of the flap and the surrounding stromal from 1 o’clock to 5 o’clock and from 7 o’clock to 9 o’clock (Fig. 3). There were no obvious corneal abnormalities between the lesions, and the epithelial cells were intact in both eyes. Consider noninfective interstitial keratitis in both eyes. On the sixth day after surgery, the patient had no complaints of discomfort, the corrected visual acuity could reach 20/20, and it could be seen that the lesions still existed in both eyes without further development and expansion (Fig. 3). After oral steroid treatment, the reexamination showed that the infiltration of the left eye was significantly reduced compared with before, the infiltration of the stromal of the right eye was also significantly limited, and the corneal confocal microscopy showed that inflammatory material deposition in the stromal layer was significantly reduced (Fig. 4). At the latest review, the corrected visual acuity of both eyes was 20/20, the corrected visual acuity was 20/20 in both eyes, the infiltrations in both eyes almost disappeared (Fig. 3), the corneal was nearly transparent, the anterior segment OCT also showed that the white infiltration was reduced, only a small amount of corneal stromal scar was left (Fig. 5), and the prognosis was good.

## Case 3

**Figure 6:**
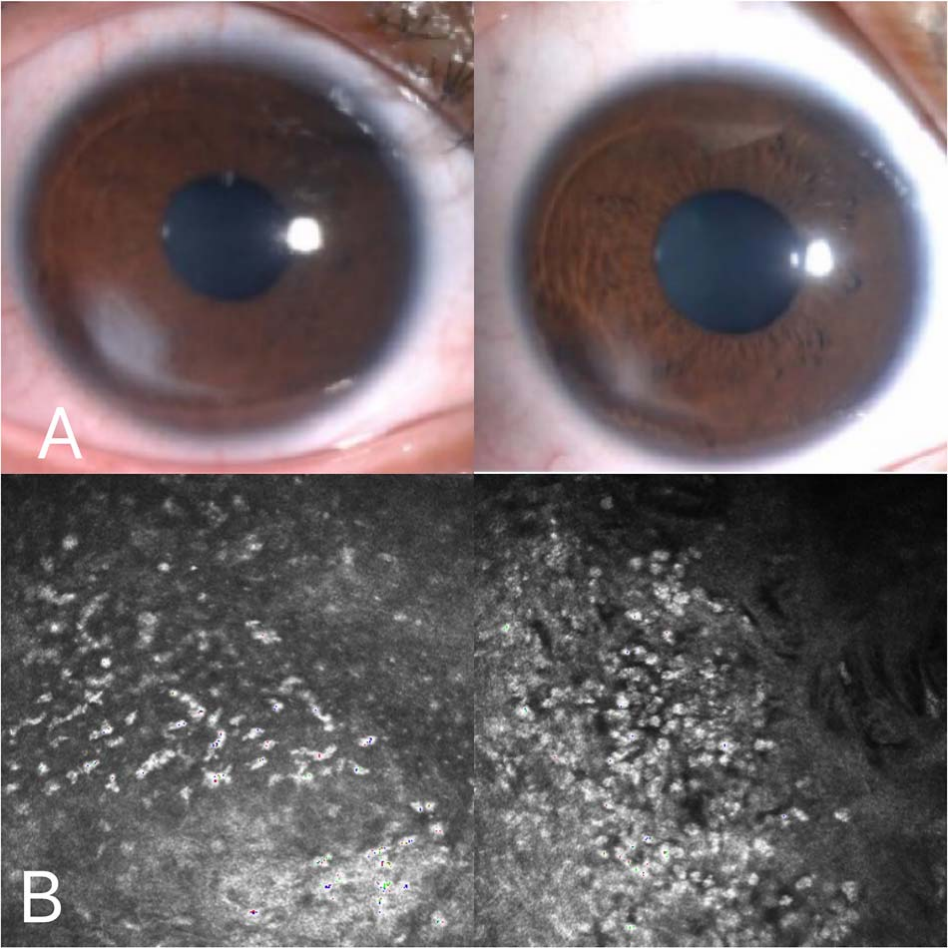
One month later after the surgery, white confluent stromal infiltrate peripheral to the flap edge, from 6 to 8 o’clock in the right eye accompanied by local epithelial defects (A); One week later after the Steroid treatment, reexamination showed that the infiltration of the right eye was alleviated (A). Corneal confocal microscopy:inflammatory cells and inflammatory exudates in the epithelial and interstitial layers but no evidence of infection (B).

**Figure 7:**
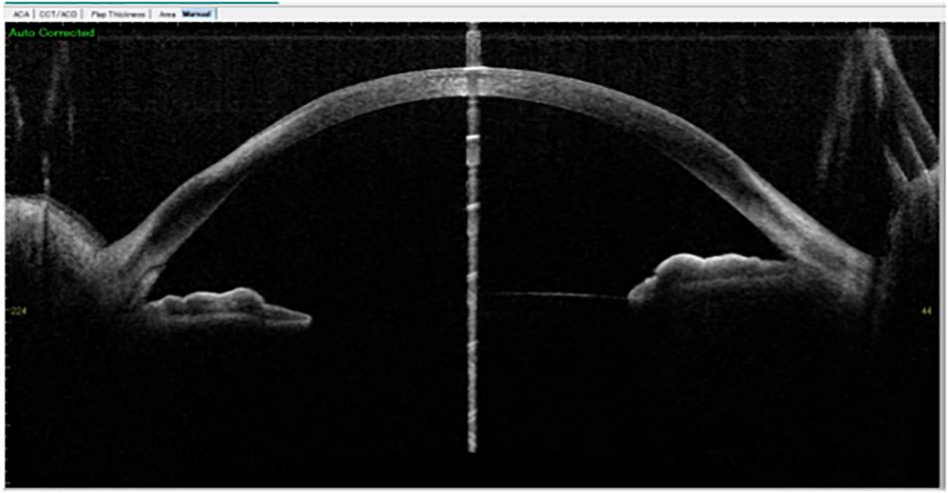
Two weeks later: Anterior segment OCT showed residual stromal scar in the stromal layer of the right eye.

A young female performed uneventful SMILE surgery in both eyes on July 2022. The corrected visual acuity in both eyes can not reach 20/20 1 week after surgery and 2 weeks after surgery, the corrected visual acuity in both eyes was only 20/25, however, there was no obvious stromal infiltration in both eyes at this stage. One month later, a white confluent stromal infiltrate peripheral to the flap edge, from 6 to 8 o’clock in the right eye accompanied by local epithelial defects (Fig. 6). Corneal confocal microscopy revealed inflammatory cells and inflammatory exudates in the epithelial and interstitial layers but no evidence of infection (Fig. 6). Steroid hormones were administered orally and locally. One week later reexamination showed that the infiltration of the right eye was alleviated (Fig. 6). Two weeks later, the infiltration was further reduced and A small amount of corneal nebula remains. Anterior segment OCT showed residual stromal scar in the stromal layer of the right eye (Fig. 7).

## Discussion

The mechanism is still unclear. Non-infective necrotizing interstitial keratitis is a rare complication of corneal refractive surgery, and its incidence is even lower in normal subjects. All three of our patients were normal persons without systemic or ocular disease, but presented with similar non-infective necrotizing infiltrative lesions of the corneal stromal layer. There have been several reports on non-infectious necrotizing interstitial keratitis after corneal refractive surgery [1]. It has been suggested that the use of non-steroidal anti-inflammatory drugs instead of steroid anti-inflammatory drugs, the abuse of local anesthetics, and autoimmune factors are related to this noninfective corneal infiltration [2]. However, none of our three patients experienced this condition.

Autoimmune factors and hypersensitivity after staphylococcal infection are currently recognized mechanisms for the occurrence of noninfective necrotizing keratitis after LASIK surgery [3]. The potential initiating events that may lead to a series of immune responses include the deposition of immune complexes, serum autoantibodies, abnormal expression of HLA-II antigens on corneal epithelium and corneal cells, vascular injury, abnormal cellular responses to corneal injury. Patient 1 had a history of travel before surgery, but there has been no report on travel and noninfective corneal infiltration after corneal refractive surgery, and whether there is a correlation remains to be studied. What was special in the medical history of patient 2 was that she had received a 9-valent vaccine against cervical cancer 3 days before surgery. We wondered whether the injection of the vaccine led to excessive inflammatory expression in the body. Although the patient denied a history of systemic autoimmune disease, laboratory tests for autoantibodies, such as an antinuclear antibody, rheumatoid factor, anti-double-stranded DNA antibody, anti-ribonucleoprotein antibody, and anti-Smith antibody, were all negative. It has previously been reported that patients with various autoimmune diseases have a high incidence of noninfective corneal infiltration after LASIK surgery [4]. Carp *et al.* [5] describe a case of a 50-year-old middle-aged woman with inflammatory bowel disease who developed stromal infiltration at and around the edges of both flaps after surgery, which resolved after treatment with local and systemic steroid therapy . Muhammad *et al.* [6] also reported the possibility of postoperative non-infective necrotizing interstitial keratitis even in patients with inactive autoimmune disease. Rodriguez-Prats *et al.* [7] reported a 36-year-old woman with chronic psoriasis who presented with stromal infiltration in the right eye with systemic acute skin lesions after LASIK, and the right eye infiltration disappeared and the systemic skin lesions resolved after steroid treatment. Geier *et al.* [8] conducted an epidemiological investigation based on whether the incidence of various autoimmune diseases after HPV vaccination was higher than that of the normal group in the VAERS database. It has been observed that autoimmune diseases such as gastroenteritis, rheumatoid arthritis, thrombocytopenia, systemic lupus erythematosus, vasculitis, alopecia, central nervous system demyelination, ovarian damage, and inflammatory bowel disease are associated with HPV vaccination. Miranda *et al.* [9] based on a large cohort of 2 million young women in France, showed that HPV vaccination may increase the risk of Guillain–Barre syndrome, especially in the first few months after vaccination in sensitivity analyses.

There is no clear explanation of the pathogenesis of tardus necrotizing corneal interstitial keratitis at present, and there are many speculations about the etiology. Morales *et al*.[10] identified epithelial defects as an important risk factor for the development of tardus necrotizing corneal interstitial keratitis. Kamiya *et al.* [11] suggested that the autoimmune response caused by staphylococcal infection was the main reason for the appearance of necrotizing interstitial keratitis. Li *et al.* [12] suggested that mechanical trauma plays an important role in the development of necrotizing interstitial keratitis. The exact cause remains to be determined.

In summary, the three cases of non-infective necrotizing interstitial keratitis after corneal refractive surgery in normal people have their characteristics. Clinicians should pay more attention to patients with a history of travel and vaccination, which also emphasizes the importance of detailed medical history. In addition, regular postoperative review and correct treatment are also very important. Not all the other three patients have a good prognosis. When no corneal infiltration occurs again but the patient has poor corrected visual acuity, the possibility of such complications should be considered, and timely diagnosis and correct treatment should be given.
